# Eosinophils Enhance Granuloma-Mediated Control of Persistent Salmonella Infection

**DOI:** 10.21203/rs.3.rs-5610725/v1

**Published:** 2025-01-03

**Authors:** Denise Monack, Daniel Butler, Blanda Di Luccia, José Vilches-Moure

**Affiliations:** Stanford University School of Medicine; Stanford University; Stanford University; Stanford University

## Abstract

*Salmonella enterica* can persist asymptomatically within tissues for extended periods. This remarkable feat is achieved through intricate host-pathogen interactions in immune cell aggregates called granulomas, wherein *Salmonella* find favorable cellular niches to exploit while the host limits its expansion and tissue dissemination. Here, using a mouse model of persistent *Salmonella* infection, we identify a host-protective role of eosinophils in control of *Salmonella* Typhimurium (*S*Tm) infection within the mesenteric lymph nodes (MLN), the main lymphoid tissue of *S*Tm persistence. Combining spatial transcriptomics and experimental manipulations, we found that macrophages responding to *S*Tm infection recruited eosinophils in a C-C motif chemokine ligand 11 (CCL11)-dependent manner and enhanced their activation. Eosinophil deficiencies increased *Salmonella* burdens, which was associated with altered granuloma size and impaired type-1 immunity in the MLN. Thus, eosinophils play a vital role in restraining *Salmonella* exploitation of granuloma macrophages at a key site of bacterial persistence.

## Introduction

Nearly 14.3 million illnesses and 136,000 deaths occur each year due to systemic infections caused by *Salmonella enterica*^1^. Host-adapted *Salmonella* serovars can persist within tissues for extended periods of time in asymptomatic hosts^2,3^. Using a chronic asymptomatic *Salmonella enterica* serovar Typhimurium (*S*Tm)infection model in mice, it has been shown that the pathogen can persist within macrophages in systemic tissues such as the spleen, liver, and mesenteric lymph nodes (MLN) for long periods of time within granulomas, with the invariant site of persistence occurring in the MLN^4,5^. Granuloma formation is believed to be a crucial immune response for controlling chronic infections by containing the bacteria to facilitate their eventual eradication and to minimize immunopathology^6,7^. Granulomas can also enable pathogen persistence and thus, furthering our knowledge of the host pathways that influence *Salmonella* granuloma formation and maintenance may inform future therapies.

Previous studies have shown that IFN-γ-dependent signaling is essential for controlling persistent *S*Tm infections^4,8^. Indeed, Goldberg et al. have shown that *S*Tm infection stimulates robust expansion of CXCR3^+^ T_H_^1^ cells that surround the granuloma cores where *S*Tm persist in nitric oxide synthase (iNOS)-producing resident and recruited macrophages^9^. Furthermore, the granulomas were surrounded by mononuclear phagocytes producing the chemokines CXCL9 and CXCL10 that primarily drive cytotoxic T cell recruitment. Interestingly, blockade of CXCR3 inhibited T_H_^1^ occupancy of CXCL9/10-dense regions and reduced activation of the T_H_^1^ cells, which led to increased *S*Tm growth^9^. Collectively, these findings suggest that type 1 immune responses contribute to granuloma formation. Intrestingly, splenic *S*Tm granuloma macrophages were recently characterized in detail, identifying a number of different subsets consisting of macrophages with both pro-inflamamtory and woundhealing phenotypes^10^. In line with these observations, Cronan *et al*.^11^ describe both type 1 and type 2 immune responses within *Mycobacterium tuberculosis* (*Mtb*) granulomas. Indeed, they found that type 2 immune signaling, mediated by *Stat6*, is required for epithelization and granuloma formation^11^. Although the molecular framework driving epithelization in *S*Tm granulomas is less understood, these structural changes have been observed outside of the granuloma core^9^. Together, these observations suggest that granuloma formation and maintenance require complex interplays between disparate immune pathways.

Building on this understanding, it has been observed that the macrophage response and subsequent formation of granulomas in response to various pathogens are typically associated with distinct macrophage polarization. During the formation of *Mtb* granulomas, the initial response and formation of granulomas is primarily driven by an M1-macrophage phenotype, characterized by a pro-inflammatory response with potent antimicrobial effects^12 13^. Over time, however, to mitigate the ongoing inflammation and associated tissue damage, M2-macrophages are recruited to the periphery of the granulomas, where they exhibit anti-inflammatory properties and contribute to tissue repair^14,15^. While this balance in immune polarization helps protect the host, it also creates a permissive niche for bacterial replication^16^. In the context of *Salmonella* infection, certain serovars express effector proteins that induce an M2-like phenotype, that further promotes a replicative niche^17,18^. For instance, Pham et al. (2020)^19^ demonstrated that the *Salmonella* effector SteE is critical for splenic granuloma formation and the creation of a permissive environment for bacterial replication by inducing IL-4Rα expression through a mechanism involving STAT3 phosphorylation^19,20^.

Here we use the persistent mouse model of chronic *S*Tm infection to investigate host immune pathways involved in pathogen persistence in MLN granulomas. Using cytokine arrays and spatial transcriptomics we show that *S*Tm infection triggers the release of the eosinophil chemoattractant CCL11 by specific macrophage populations in MLN during the chronic phase of infection. We show that CCL11 is required for recruitment of eosinophils, which have traditionally been considered end stage effector cells linked to parasitic infections and pathological type 2 disease states such as asthma and allergies, to *S*Tm-infected MLN. In addition, eosinophils have previously been shown to contribute to the antimicrobial defense during chronic *Mtb* infection and localize to the periphery of lung granulomas in humans, non-human primates and zebra fish^21^. However, eosinophils have not been demonstrated to play a role in *Mtb* granuloma development and/or maintenance^21^. In contrast, eosinophils suppress local TH1 responses in the presence of *Helicobacter pylori* along the gastrointestinal tract^21,22^. Here, we show that eosinophils are part of the antimicrobial defense against *Salmonella* during persistent infection and that eosinophil-deficient mice influence local myeloid responses and granuloma phenotype. Using Xenium single cell *in situ* analysis we show that eosinophils are closely associated with MLN granuloma responses. Overall, our results demonstrate that eosinophils are crucial for maintaining granuloma integrity within the infected MLN and are important for maintaining pathogen levels during persistent *Salmonella* infection in the MLN. Together, these findings support a model where eosinophils actively shape the local immune landscape and contribute to a local, organized response that is essential for pathogen control in the MLN.

## Results

### Chronic STm infection triggers a robust CCL11 response in the MLN

**Figure 1:**
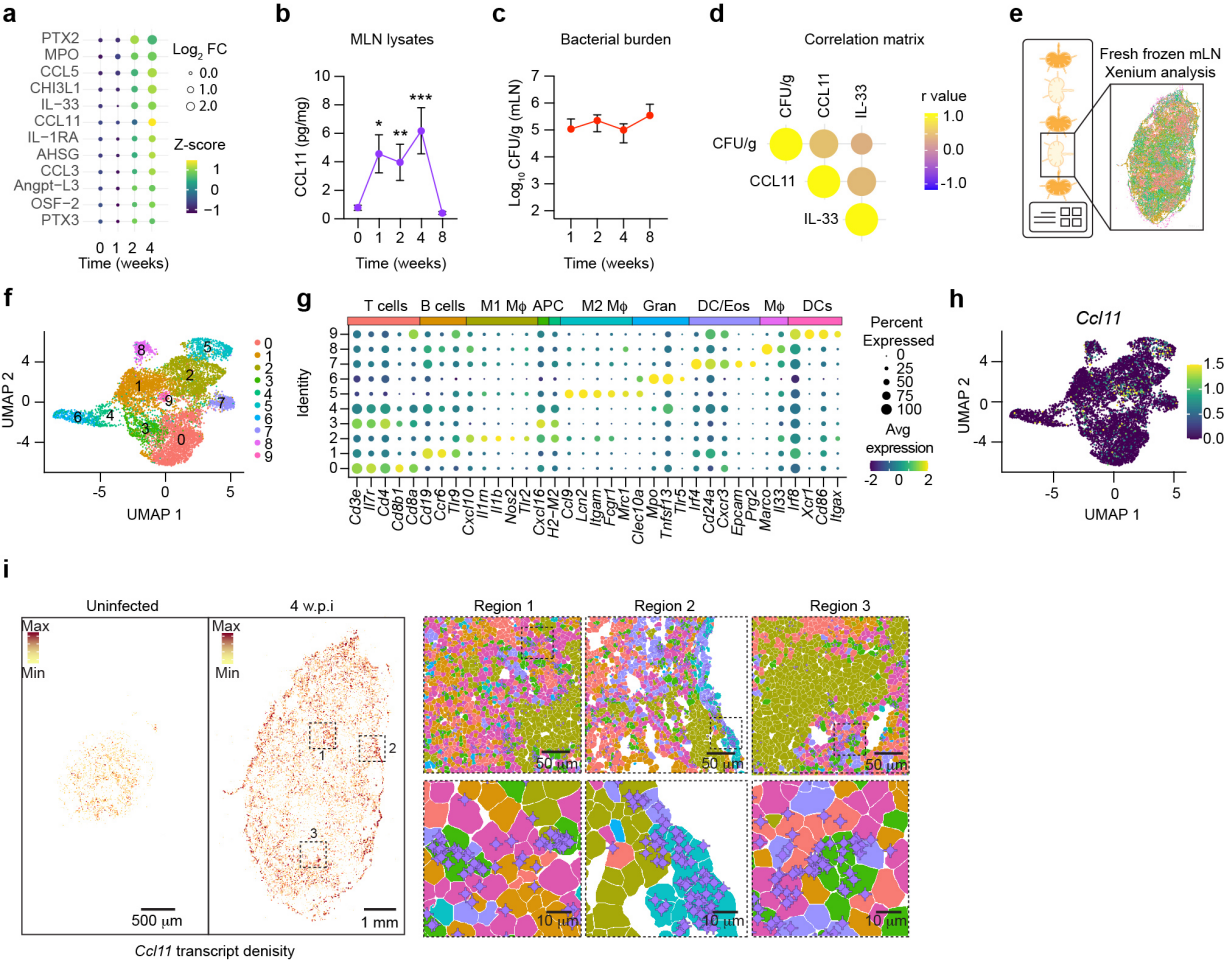
Cytokine and spatial transcriptomics analyses identify CCL11 and macrophage populations that correlate with *S*Tm persistence. 129×1/SvJ mice were orally infected with wild-type STm SL1344 (10^8^ CFUs) and monitored for 8 weeks. (**a**) Heatmap showing significantly regulated soluble mediators in *S*Tm-infected MLN supernatants across a panel of 111 at analytes at 1-, 2- and 4 w.p.i. compared to uninfected controls. Data represent pooled samples from n = 10 mice per time point from 2 independent experiments. (**b**) CCL11 levels in STm-infected MLN supernatants at 1-, 2-, 4-, and 8 w.p.i. compared to uninfected controls by ELISA (n = 10 mice per time point from two independent experiments). (**c**) Bacterial burden in STm-infected MLN at 1-, 2-, 4- and 8 w.p.i. from n = 10 mice per time point. (**d**) Heat-map of a correlation matrix between MLN bacterial levels and MLN tissue levels of CCL11 and IL-33. Data from uninfected controls and STm-infected mice at 1-, 2-, 4- and 8 w.p.i. (n = 10 mice per time point). (**e**) Schematic representation of Xenium single-cell in situ analysis of MLN tissue sections from uninfected controls and STm-infected mice at 4 w.p.i MLN were sectioned from 8 infected and 2 uninfected mice from 3 independent experiments. (f) UMAP plot based on unsupervised clustering of Xenium single cell in situ analysis of MLN from uninfected controls and STm-infected mice at 4 w.p.i. Cluster 0 = salmon, cluster 1 = beige, cluster 2 = olive green, cluster 3 and 4 = green, cluster 5 = cyan, cluster 6 = light blue, cluster 7 = purple, cluster 8 = pink and cluster 9 = magenta. (**g**) Heatmap showing the top 1–5 differentially expressed genes within each cluster from UMAP analysis in Fig. 1f. (Cut-off; Log_2_ FC = 1.41, *P* < 0.05). (**h**) UMAP plot showing the expression of *Ccl11*. Results obtained from 10 mice, 8 infected and 2 uninfected mice from 3 independent experiments. (**i**) Image showing *Ccl11* transcript density in MLN from uninfected controls and *S*Tm-infected mice at 4 w.p.i. *Ccl11* enriched regions are outlined. Purple symbols indicate *Ccl11* transcripts, and segmented cells are colored according to UMAP cluster identities from Fig. 1F. Representative images is shown from a total of 10 mice. Scale bars = 500 mm and 1 mm; insets = 50 mm and 10 mm. Data is presented as Means ± SEM and were analyzed by Kruskal-Wallis test with Dunn’s correction (A, B), non-parametric Pearson correlation (D) and Wilcoxon rank-sum test (G). * = *P* < 0.05, ** = *P* < 0.01, *** = *P* < 0.001.

MLN serve as a major reservoir of persistent *S*Tm colonization^4^, although the underlying mechanisms remain elusive. To better understand how host responses both control and permit *S*Tm survival over prolonged periods, we orally infected 129×1/svJ (129 WT) mice and monitored cytokines, chemokines and soluble mediators in MLN supernatants across 4 weeks (Fig. 1a, **Exteded data 1a**). Analysis using a broad cytokine array detected minimal changes in analytes during the first week post-infection (w.p.i) compared to uninfected controls. By two weeks, however, significant elevations were noted in pro-inflammatory mediators lilnked to neutrophil responses such as MPO, PTX2 and CHI3L1, with further increases in pro-inflammatory cytokines/proteins, chemokines and proteins involved in tissue repair and angiogenesis by four w.p.i. (Fig. 1a, **Exteded Data Fig. 1a**). Interestingly, levels of eosinophil-related mediators of chemotaxis and activation like CCL11, CCL5 and IL-33 increased by four w.p.i.^23,24^ (Fig. 1a, **Exteded Data Fig. 1b, c**). We confirmed that the MLN levels of CCL11 were significantly higher 1–4 w.p.i compared to the levels prior to infection. Importantly, the increased CCL11 levels significantly correlated with increased bacterial loads in the MLN (Fig. 1B–D, **Extended Data Fig. 1D**). In contrast to the MLN, CCL11 levels in the spleen remained unchanged compared to uninfected mice despite significant *S*Tm burdens, highlighting a tissue-specific response (**Extended Data Fig. 1e, f**). These findings suggest an intricate cytokine response that may drive eosinophil recruitment during persistent *S*Tm infection of the MLN.

### Spatial transcriptomics analysis of chronically STm-infected MLN shows distinct macrophage profiles, some localized to granulomas and others expressing Ccl11.

To gain insights into cellular sources of CCL11 and their location in the MLN, we employed Xenium single-cell *in situ* analysis to provide high-resolution gene expression mapping in the MLN of both uninfected controls and *S*Tm-infected mice at 4 w.p.i. (Fig. 1e). Automatic segmentation revealed a range of 19,000–206,667 analyzed cells across 10 samples, with a median transcript count per cell ranging from 34 to 203. To understand the general transcriptomic landscape, we normalized the data, performed batch correction from the three independent runs and performed unbiased clustering, identifying 10 distinct UMAP clusters based on gene expression profiles inferred from previously published scRNA-seq datasets^10,25–27^.

The majority of cells were grouped into two dominant clusters (Fig. 1f): cluster 0, enriched for T cell markers (e.g., Cluster of differentiation (*Cd*) *3 epsilon, Cd4, Cd8a, Cd8b1*), and cluster 1, enriched for B cell markers (e.g., *Cd19, Ccr6* (C-C chemokine receptor type 6) and *Tlr9* (Toll-like receptor 9)). Five clusters represented various macrophage and dendritic cell populations. For example, Cluster 2 showed increased expression of M1-like macrophage markers (e.g. *Il1b* (Interleukin-1 beta), *Nos2* (Nitric oxixide synthase 2) and *Cxcl10* (C-X-C motif chemokine ligand 10, also know as Interferon-gamma-induced protein 10)), while cluster 5 had increased expression of M2-like macrophage markers (e.g., *Fcgr1* (FC-gamma receptor 1), *Clec10a* (C-type lectin domain family 10 member A) and *Mrc1* (Mannose receptor, C type 1)). Cluster 3 had high expression of *H2-M2* (histocompatibility 2, M region locus) and *Cxcl16* (C-X-C motif chemokine ligand 16), a marker of inflammation. Cluster 4 also expressed *H2-M2* and *Cxcl16* and had high expression of *Tnfsf13* (Tumor necrosis factor ligand superfamily member 13B, also known as BAFF). Cluster 8 was defined by high expression of the macrophage marker *Marco*, along with intermediate expression of *Il33* (Interleukin-33) and *Mrc1*. Cluster 9 was defined by dendritic cell markers (e.g., *Itgax, Irf8, Xcr1* and *Cd86*). Additionally, two granulocyte-like clusters were identified: cluster 6, was delineated by high expression of neutrophil-associated genes (e.g., *Mpo* (Myeloperoxidase), *Tlr5* (Toll-like receptor 5)), and cluster 7, contained transcripts associated with eosinophils (e.g., *Prg2* (eosinophil major basic protein), *Cd24a* (a glycosylphosphatidylinositol-anchored surface protein) (Fig. 1f, g). Comparing the gene-expression profiles of uninfected and infected mice revealed a notable increase in cells belonging to clusters 2, 5, 7 and 8 in the infected mice (**Extended Data Fig. 1g**).

Spatial analysis highlighted distinct organization patterns for most clusters within MLN. In the infected MLN, M1- and M2-like macrophages were localized differently; M2-like macrophages (cluster 5) were primarily found in the capsule and subcapsular sinus (SCS), and M1-like macrophages (cluster 2) formed dense, granuloma-like structures throughout the MLN at 4 w.p.i. (**Extended Data Fig. 1h, i**). The eosinophil cluster (cluster 7) was primarily observed in proximity to the M1-structures and to the SCS while the remaining myeloid clusters localized across the infected MLN (**Extended Data Fig. 1h**). To understand if these M1-granuloma structures might be influenced by cannonical signaling cascades we localized *Ifng* transcripts in the infected MLN. Indeed, we observed an enrichment of *Ifng* transcripts in close proximity to M1-granuloma structures (**Extended Data Fig. 1J**). This aligns with the known role of IFN-g in controlling chronic *S*Tm infection^4,8^.

Since CCL11 levels correlated with pathogen levels in the MLN, we mapped *Ccl11* transcripts in *S*Tm-infected MLN 4 w.p.i.. Our additional findings confirm elevated *Ccl11* transcripts in the MLN (Fig. 1h). Subcluster analysis of *Ccl11* expressing cells revealed that *Ccl11* was primarily expressed by two distinct macrophage/monocyte clusters. These included M2-like macrophages expressing *Mrc1, Clec10a* and *Fcgr1* and a cluster of monocyte/macrophages expressing *Cd14* and *Trem2* (Triggering receptor expresson myeloid cells-2), signifying a type of monocyte/macrophage with enhanced phagocytic and anti-inflammatory properties^28,29^ (Fig. 1h, **Extended Data Fig. 1k**). The presence of CCL11 in these populations was confirmed via western blot post-cell sorting (**Extended Data Fig. 1l**). Finally, spatial mapping showed distinct *Ccl11* transcript enrichments across three regions in MLN tissue sections (Fig. 1i). Region 1, encompassing the T cell zone (cluster 0 cells), and region 3, near the periphery of granuloma M1-macrophages (cluster 2 cells), showed *Ccl11* transcripts primarily in *Cd14*^*+*^ *Trem2*^*+*^ (cluster 3) cells. However, in region 2, located in the MLN capsule/subcapsular sinus area, *Ccl11* was mainly expressed in M2-like macrophages (cluster 5 cells) (Fig. 1i). This pattern underscores the involvement of specific macrophage subsets in *Ccl11* expression, which may play a role in directing localized immune responses during persistent *S*Tm colonization.

### CCL11 neutralization leads to specific inhibition of eosinophil recruitment and elevated MLN STm burdens

**Figure 2:**
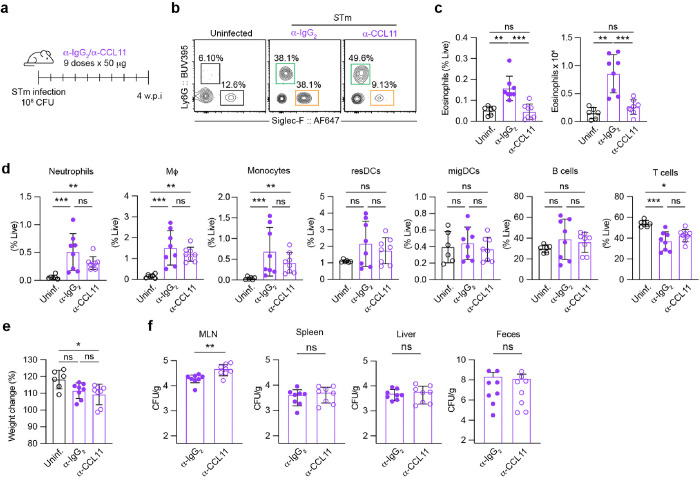
Targeted CCL11 neutralization depletes eosinophils and increases bacterial burdens in MLN. (a) Schematic of the CCL11-neutralization approach. Mice were orally infected with STm (10^8^ CFUs) and treated with either anti-CCL11 antibodies or anti-IgG_2_ antibodies starting at day 3 post infection and continuing every 3 days until day 28. (**b**) Representative FACS plots of neutrophils and eosinophils isolated from MLN in *S*Tm-infected anti-IgG_2_ treated mice and anti-CCL11 treated mice at 4 w.p.i, compared to uninfected controls. (**c**) Flow cytometry quantification of the frequency of live and absolute numbers of eosinophils isolated from MLN of *S*Tm-infected mice treated with either anti-IgG_2_ treated mice (purple closed circles) or anti-CCL11 treated mice (purple open circles) at 4 w.p.i, compared to uninfected controls (black open circles). (**d**) Quantification of the frequency of neutrophils, macrophages, monocytes, resident dendritic cells (DCs), migratory DCs, T cells and B cells isolated from MLN in *S*Tm-infected anti-IgG_2_ treated mice and anti-CCL11 treated mice at 4 w.p.i, compared to uninfected controls. (**e**) Percentage of body weight variation in *S*Tm-infected anti-IgG_2_ treated mice and anti-CCL11 treated mice at 4 w.p.i, compared to uninfected controls compared to day 0. (**f**) Quantification of MLN-, splenic-, liver and fecal bacterial levels in *S*Tm-infected anti-IgG_2_ treated mice and anti-CCL11 treated mice at 4 w.p.i.. Data are presented as Means ± SEM from *n* = 6–8 mice per group from two independent experiments. Data was analyzed by Kruskal-Wallis U-test (C-E) or Mann-Whitney U-test (F). ns = non-significant, * = *P* < 0.05, ** = *P* < 0.01, *** = *P* < 0.001.

To evaluate the impact of the eosinophil-related chemokine CCL11 during *S*Tm infection, we administered a CCL11 neutralizing antibody or an isotype control antibody to mice three days post-infection, with subsequent doses every 3 days until sacrifice at 4 w.p.i.^30^ (Fig. 2a). The neutralization notably diminished eosinophil numbers in the MLN of *S*Tm-infected mice (Fig. 2b, c, **Exteded Data Fig. 2a**). In contrast, CCL11 neutralization did not impact eosinophil numbers in the spleen, supporting the tissue-specific role of CCL11 in the MLN (**Extended Data Fig. 2c**). In addition, we characterized the effects of CCL11 neutralization on other myeloid cells and found that there was no impact on neutrophil, monocyte, dendritic cell or macrophage numbers (Fig. 2d, **Extended Data Fig. 2b**). Furthermore, we found that while CCL11 neutralization did not affect the survival or weight of the mice (Fig. 2e), it resulted in increased *S*Tm levels specifically in the MLN (Fig. 2f), as there were no major changes in *S*Tm levels in the spleen, liver or feces compared to isotype control-treated mice (Fig. 2f). Collectively, these results highlight the protective role of CCL11 and suggest a role for eosinophils in limiting *S*Tm levels during chronic infection of the MLN.

### Unique phenotypic characteristics of MLN eosinophils during persistent STm infection

**Figure 3:**
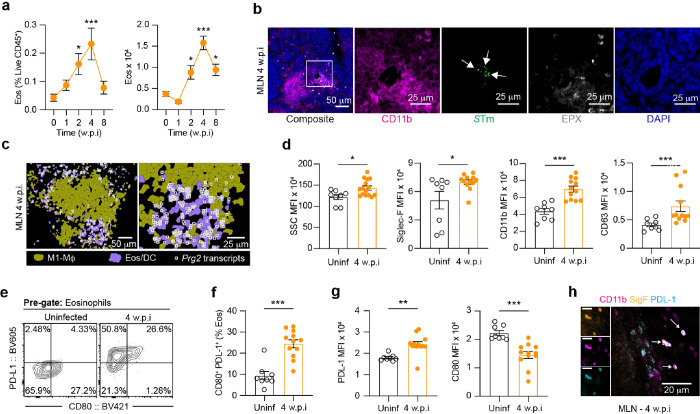
MLN eosinophils are activated and localize to the periphery of *S*Tm-containing granuloma structures. 129×1/svJ mice were orally infected with *S*Tm (10^8^ CFUs) and monitored for up to 8 w.p.i. (**a**) Quantification of the frequency of live eosinophils and absolute number of eosinophils at 1-, 2-, 4- and 8 w.p.i., compared to uninfected controls. (**b**) Immunofluorescence staining of MLN tissue sections from *S*Tm-infected mice showing eosinophils (grey) localized at the periphery of CD11b+ granulomas (magenta) containing *S*Tm (green). Arrows highlighting *S*Tm staining. Representative staining from *n* = 5 mice, with at least 2 tissue sections per mouse. Scalebars = 50 mm; insets = 25 mm. (**c**) Xenium analysis showing *Prg2* transcripts (white symbols) within segmented eosinophils in the periphery of segmented M1-Macrophages (Mf). Segmented cells are colored according to UMAP cluster identities from Fig. 1F. Representative images is shown from a total of 10 mice. Scalebars = 50 mm; insets = 25 mm. (**d**) Quantification of mean fluorescent intensity (MFI) for activation markers on MLN eosinophils isolated from uninfected controls and *S*Tm-infected mice at 4 w.p.i. showing de-novo granularity (SSC-Area), maturation (Siglec-F), migration (CD11b) and degranulation (CD63). See also Extended Data Fig. 3h. (**e**) Representative FACS plots of PD-L1 and CD80 expression on eosinophils isolated from MLN from uninfected controls and *S*Tm-infected mice at 4 w.p.i.. (**f**) Quantification of the frequency of CD80^+^ PD-L1^+^ eosinophils isolated from the MLN in uninfected controls and *S*Tm-infected mice at 4 w.p.i.. (**g**) Quantification of the mean fluorescent intensity of PD-L1 and CD80 on eosinophils isolated from MLN from uninfected and *S*Tm-infected mice at 4 w.p.i.. (**h**) Immunofluorescent staining of *S*Tm-infected MLN at 4 w.p.i showing CD11b, Siglec-F and PD-L1 triple positive eosinophils. Scale bar = 20 mm. Data are presented as Means ± SEM from 8 – 12 mice from at least 3 experiments and was analysed by Kruskal-Wallis test with Dunn’s correction (B) or Mann Whitney-U test (D, G, H). ns = non-significant, * = p < 0.05, ** = p < 0.01, *** = p < 0.001

Given the significant impact of CCL11 neutralization on eosinophil recruitment and subsequent bacterial levels, we delved deeper into the dynamics of the eosinophil response during the first eight weeks of persistent *S*Tm infection. Our kinetic analysis revealed a notable increase in MLN eosinophils by 2 w.p.i., reaching a peak at 4 w.p.i., and sustaning elevated levels up to 8 w.p.i. (Fig. 3a). This rise in eosinophil numbers closely aligns with the tissue levels of CCL11 (**Extended Data Fig. 3a**). Immunofluorescence staining showed that eosinophils (detected with eosinphil-specific marker, EPX (Eosinophil peroxidase)) predominantly localize around *S*Tm containing CD11b^+^ foci, which are indiciative of granuloma structures (Fig. 3b). This pattern of eosinophil distribution and activity is underscored by our spatial transcriptomic data, highlighting the prominence of *Prg2* positive eosinophils around these granulomatous regions (Fig. 3c).

Our findings demonstrate that during persistent *S*Tm infection, eosinophils predominantly accumulate in the MLN, while their numbers significantly decrease in the blood and spleen of mice during the 4 weeks of infection (**Extended Data Fig. 3b-e**), suggesting recruitment from systemic hemopoietic sites such as the bone marrow, blood and spleen. Diverse eosinophil populations identified through recent single-cell transcriptomic analyses underscore their functional heterogeneity across different tissues^27^. Previous studies have shown that activated eosinophils express elevated levels of specific markers such as *de novo* granulation (e.g., side scatter (SSC)-area), maturation (e.g., Siglec-F), migration (e.g., CD11b), and degranulation (e.g., CD63) (REF). We show that eosinophils isolated from *S*Tm-infected mice had increased levels of all activation markers compared to uninfected controls (Fig. 3d, **Extended Data Fig. 3f, g**). In addition, eosinophils from mice infected with *S*Tm for 4 weeks exhibited a distinct activation profile, with the majority of cells expressing high levels of PD-L1, with a subset of PD-L1 positive cells also expressing CD80 (Fig. 3e–h). This subset of activated eosinophils has previously been described to have potent antimicrobial and immunoregulatory functions in the gastrointestinal tract^27^. Taken together, this observation underscores the significant presence of eosinophils around *Salmonella*-infected macrophages within the MLN, suggesting that they may have antimicrobial and/or immunoregulatory roles during persistent infections.

### Eosinophils contribute to antimicrobial defense against STm in persistent infections

**Figure 4:**
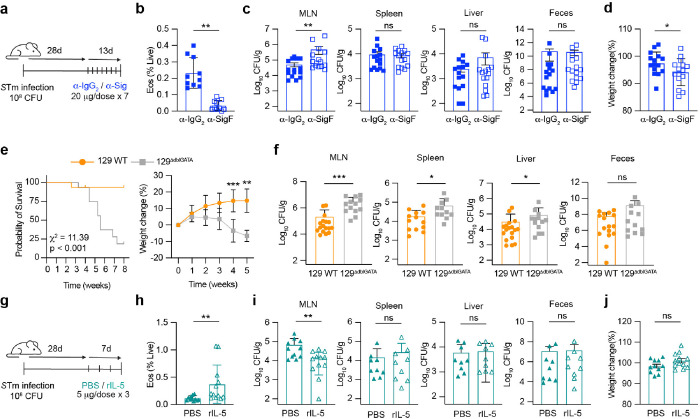
Eosinophil-deficient mice are more susceptible to persistent *S*Tm infection (a) Schematic of eosinophil depletion using anti-Siglec F antibodies. Mice were infected with STm for 4 weeks before receiving seven doses of either anti-IgG_2_ or anti-Siglec F antibodies every two days prior to sacrifice on day 41. (**b**) Quantification of the frequency of live MLN isolated from *S*Tm-infected mice treated with anti-IgG_2_ (blue filled squares) or anti-Siglec F (blue open squares) at 41 days post infection. (FACS plot in Extended Data Fig. 4a). (**c**) Quantification of bacterial levels in MLN, spleen, liver and feces in *S*Tm-infected mice treated with anti-IgG_2_ or anti-Siglec-F at 4 w.p.i.. (**d**) Percentage of body weight variation in *S*Tm-infected mice treated with isotype control or anti-Siglec F antibody compared to first antibody treatment on day 28. (**e**) Kaplan-Mayer survival curve comparing *S*Tm-infected 129 WT mice (orange) and genetically eosinophil-deficient 129^DdblGATA1^ mice (grey) over 8 weeks along with body weight variation in the same groups. (**f**) Quantification of bacterial levels in MLN, spleen, liver and feces in 129 WT and eosinophil-deficient 129^DdblGATA1^ mice at 4 w.p.i. (**g**) Schematic of transient eosinophilia mediated by rIL-5 treatment. 129xsvJ mice were infected for for 4 weeks before recieving 3 doses of rIL-5 or PBS every two days prior to sacrifice on day 35 (5 w.p.i). (**h**) Quantification of the frequency of live MLN eosinophils isolated from the MLN of STm-infected PBS-treated mice (closed teal triangles) and rIL-5-treated mice at 5 w.p.i (open teal triangles). (FACS plot in Extended Data Fig. 4h) (**i**) Quantification of bacterial levels in the MLN, spleen, liver and feces at 5 w.p.i. (**j**) Percentage of body weight variation in STm-infected PBS- and rIL-5-treated mice compared to first treatment at 4 w.p.i.. Data are presented as Means ± SEM from 8–15 mice from at least 3 experiments and was analysed by Mann Whitney-U test (C-E, K-M), Kruskal-Wallis with Dunn’s correction (G) or Mantel-Cox analysis. ns = non-significant, * = p < 0.05, ** = p < 0.01, *** = p < 0.001

Having established the recruitment and activation of eosinophils in the MLN during persistent infection, we next explored their functional role in controlling *S*Tm. To determine the impact of eosinophils on infection outcomes, we injected mice that had been infected with *S*Tm for 28 days with anti-Siglec-F antibodies to deplete eosinophils or an isotype IgG_2_ control antibody (Fig. 4a). The anti-Siglec-F antibody depletion led to a dramatic decrease in eosinophil levels compared to mice treated with the isotype control antibody (Fig. 4b, **Extended Data Fig. 4a**). Notably, anti-Siglec-F treated mice exhibited elevated MLN bacterial burden while CFUs in the spleen, liver and feces remained unchanged (Fig. 4c), which is similar to what we saw with CCL11 neutralization (Fig. 2f). Although the transient depletion of eosinophils did not impact survival, there was a small weight decrease in this group compared to isotype controls (Fig. 4d). The observation that eosinophil depletion increases bacterial burdens in the MLN, but not in other organs, underscores their specific role in controlling *S*Tm within the MLN during persistent colonization.

### Eosinophil-deficient mice are susceptible to persistent STm infection

To assess whether the absence of eosinophils influences acute *S*Tm infection dynamics, we examined the susceptibility of eosinophil-deficient DdblGATA1 mice using a C57BL/6 background, which is known for its heightened susceptibility to *S*Tm infection. Our investigations did not reveal any significant differences in weight loss, survival, or the spread of bacteria systemically during the acute phase of typhoid-like disease (**Extended Data Fig. 4b, c**). These results suggest that eosinophils do not significantly influence the outcomes of acute *Salmonella* infection.

Expanding on this, we backcrossed ΔdblGATA1 mice onto the 129×1/svJ background to study persistent infection dynamics (**Extended Data Fig. 4d**). There were no baseline differences between 129 WT and eosinophil-deficient 129^ΔdblGATA1^ mice (**Extended Data Fig. 4e**). 129 WT and eosinophil-deficient 129^ΔdblGATA1^ mice were infected and monitored over a period of eight weeks. By 4 w.p.i., eosinophil-deficient 129^ΔdblGATA1^ mice exhibited significant weight loss, and by 6 w.p.i., there was notable mortality, with a median survival of 37 days (Fig. 4e). Histopathological studies were performed to examine the consequences of *S*Tm infection on tissue integrity, inflammation, and lesion formation in 129 WT and 129^ΔdblGATA1^ mice at 4 w.p.i. Differences in histopathological lesions between WT and eosinophil-deficient mice were primarily observed in the cecum and colon (**Extended Data Fig. 4f**). Closer investigation of the MLN revealed only minor changes with both groups having an expansion of plasma cells, indicative of an antigenic response compared to uninfected mice. Interestingly, 129 WT mice showed aggregates of histocytes that resemble organized granulomas which were smaller in the eosinophil-deficient 129^ΔdblGATA1^ mice (**Extended Data Fig. 4g**). Strikingly, we found that eosinophil-deficient 129^ΔdblGATA1^ mice had significantly elevated bacterial loads in the MLN and increased bacterial burden in the spleen and liver at 4 w.p.i., compared to WT mice (Fig. 4f). This pronounced susceptibility highlights the crucial role of eosinohpils in controlling bacterial persistence and possibly modulating the immune response within the MLN during persistent *Salmonella* colonization.

### Transient eosinophilia enhances antimicrobial defenses during persistent STm infection

To further explore the role of eosinophils in managing persistent *S*Tm infection, we specifically investigated the effects of bolstering eosinophil numbers in chronically infected mice. We administered intraperitoneal (IP) injections of recombinant IL-5, a cytokine that stimulates proliferation and maturation^31^. Post 28 days of *S*Tm infection, mice were administered three doses of rIL-5 or a PBS placebo (Fig. 4g). The rIL-5 treatment notably increased eosinophil numbers in the MLN, doubling their presence compared to the control group (Fig. 4h, **Extended Data Fig. 4h)**. This increase did not affect overall pathology or weight but led to a significant reduction in MLN bacterial levels (Fig. 4i, j), highlighting the localized antimicrobial role of eosinophils. In contrast, bacterial levels in other organs such as the spleen and liver, and in the feces remained unchanged (Fig. 4i), pointing to the specific impact of eosinophils within the MLN during persisent *Salmonella* infection.

### Eosinophil-deficient mice have altered myeloid responses and higher intracellular STm burdens

Previous research has demonstrated the role of eosinophils in suppressing T_H_1 immune responses in *Helicobacter pylori*-infected stomachs^22^. In contrast, in other infectious conditions, such as *Trichuris muris* infection of the MLN, eosinophils appear to promote local T_H_2 responses through the release of IL-4^32^. To examine whether WT and eosinophil-deficient 129^ΔdblGATA1^ mice have different T_H_1 or T_H_2 immune responses in the MLN, we profiled type 1 and type 2 T lymphocyte responses using surface markers (CD4 and CD8) and canonical transcription factors such as Tbet for T_H_1, GATA3 for T_H_2, and Foxp3 for Treg responses by flow cytometry (**Extended Data Fig. 5a, b**). Our findings show no significant differences in T_H_1, T_H_2 or Treg cell populations between 129 WT and eosinophil-deficient 129^ΔdblGATA1^ mice, suggesting that eosinophils do not noticeably affect the T cell response during *S*Tm infection of the MLN.

**Figure 5:**
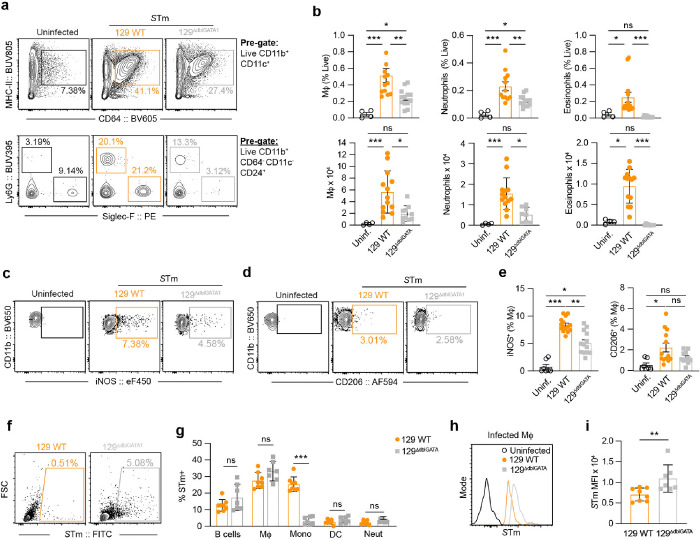
Eosinophil-deficient mice have altered myeloid responses and higher intracellular STm burdens. Eosinophil-deficient 129^DdblGATA1^ mice and 129 WT mice were orally infected with STm (10^8^ CFUs) for 4 weeks followed by flow cytometric analysis of myeloid and lymphoid populations in the MLN. (**a**) Representative FACS plots showing macrophages (Mf), neutrophils and eosinophils isolated from MLN of uninfected controls and *S*Tm-infected 129 WT and 129^DdblGATA1^ mice at 4 w.p.i. (**b**) Quantification of the frequency and absolute numbers of macrophages (Mf), neutrophils and eosinophils isolated from MLN of *STm-infected* 129 WT (orange closed circles) and 129^DdblGATA1^ mice (grey closed squares) at 4 w.p.i compared to uninfected controls (black open circles). (*n* = 4–13 mice per group from at least two separate experiments). See also Extended Data Fig. 5. (**c-d**) Representative FACS plots showing iNOS+ and CD206+ macrophages isolated from the MLN of *S*Tm-infected 129 WT and 129^DdblGATA1^ mice at 4 w.p.i compared to uninfected controls. (**e**) Quantification of the frequency of iNOS+ and CD206+ macrophages isolated from the MLN of *S*Tm-infected 129 WT (orange closed circles) and 129^DdblGATA1^ (grey closed squares) mice at 4 w.p.i., compared to uninfected controls (black open circles) from *n* = 6–14 mice per group from 3 independent experiments. (**f**) Representative FACS plots of *S*Tm-infected cells isolated from the MLN of STm-infected 129 WT and 129^DdblGATA1^ mice. (**g**) Quantification of major cellular populations positive for *S*Tm, isolated from *S*Tm-infected MLN, shown as percentages of all *S*Tm-infected cells. Data from *n* = 7–8 mice from two experiments. (**h**) Representative flow cytometry histogram of intracellular *S*Tm staining in macrophages (Mf) isolated from STm-infected MLN of 129 WT (orange) and 129^DdblGATA1^ (grey). Uninfected controls (black) served as a negative control. (**i**) Quantification of mean fluorescent intensity (MFI) for intracellular *S*Tm in Mf isolated from *S*Tm-infected MLN of 129 WT and 129^DdblGATA1^ mice. Data are presented as Means ± SEM and was analysed by Mann-Whitney U-test (B, F, G) Kruskal-Wallis with Dunn’s correction (A, D), and Mantel-Cox analysis (A). ns= non-significant* = p < 0.05, ** = p < 0.01, *** = p < 0.001

Based on our findings that eosinophils are key in controlling bacterial burden in the MLN, we explored their effects on different myeloid cells within the MLN of 129 WT and 129^ΔdblGATA1^ mice. Our analyses revealed that the absence of eosinophils led to decreased numbers of neutrophils and macrophages, while the numbers of monocytes or dendritic cells remained unchanged (Fig. 5a, b, **Extended Data Fig. 5c, d**). Further investigations in the spleen of both mouse genotypes showed no significant differences in myeloid responses, highlighting that the impact of eosinophils is specific to the MLN during persistent STm colonization (**Extended Data Fig. 5e**).

Since *S*Tm persist in iNOS^+^ macrophages within mouse tissue^9^ and replicate within a subset of bone marrow-derived macrophages expressing the M2-macrophage marker *Mrc1* (CD206)^16^, we assessed the influence of eosinophils on these macrophage populations in the MLN. We found that iNOS^+^ macrophages were less frequent in eosinophil-deficient mice compared to their WT counterparts, although the numbers of CD206 + macrophages did not differ significantly between the groups (Fig. 5c–e). Additionally, phenotypic assessments showed elevated expression of CD14, MHC-II, and CXCL9 on iNOS + macrophages, indicating a canonically activated M1-like macrophage state (**Extended Data Fig. 5f**). In contrast, CD206^+^ macrophages exhibited increased expression of another M2-marker, CD301, underscoring the distinct immunological roles of these macrophage subsets during *S*Tm infection (**Extended Data Fig. S5f**).

Based on the critical roles of neutrophils and macrophages in combating *S*Tm infections, we posited that their reduced numbers in eosinophil-deficient mice might account for increased bacterial loads observed in these animals (Fig. 4f). To address this idea, we analyzed *S*Tm infection within various immune cells using a previously described *S*Tm strain that expresses tdTomato when it is intracellular^9^. We found that macrophages were the predominant cell that harbored *S*Tm in both mouse backgrounds. However, the eosinophil-deficient mice had fewer *S*Tm-infected monocytes (Fig. 5f, g). In addition, the mean fluorescent intensity (MFI) of *S*Tm within macrophages from 129^ΔdblGATA1^ mice was significantly higher compared to 129 WT mice (Fig. 5 h, i), suggesting heightened intracellular bacterial replication within macrophages in the absence of eosinophils. These findings illustrate the crucial role of eosinophils in modulating myeloid cell responses to persistent *S*Tm colonization and influencing how STm is distributed across distinct cell populations.

### Eosinophils influence MLN granuloma immune cell composition, size, and location of STm-containing macrophages

**Figure 6:**
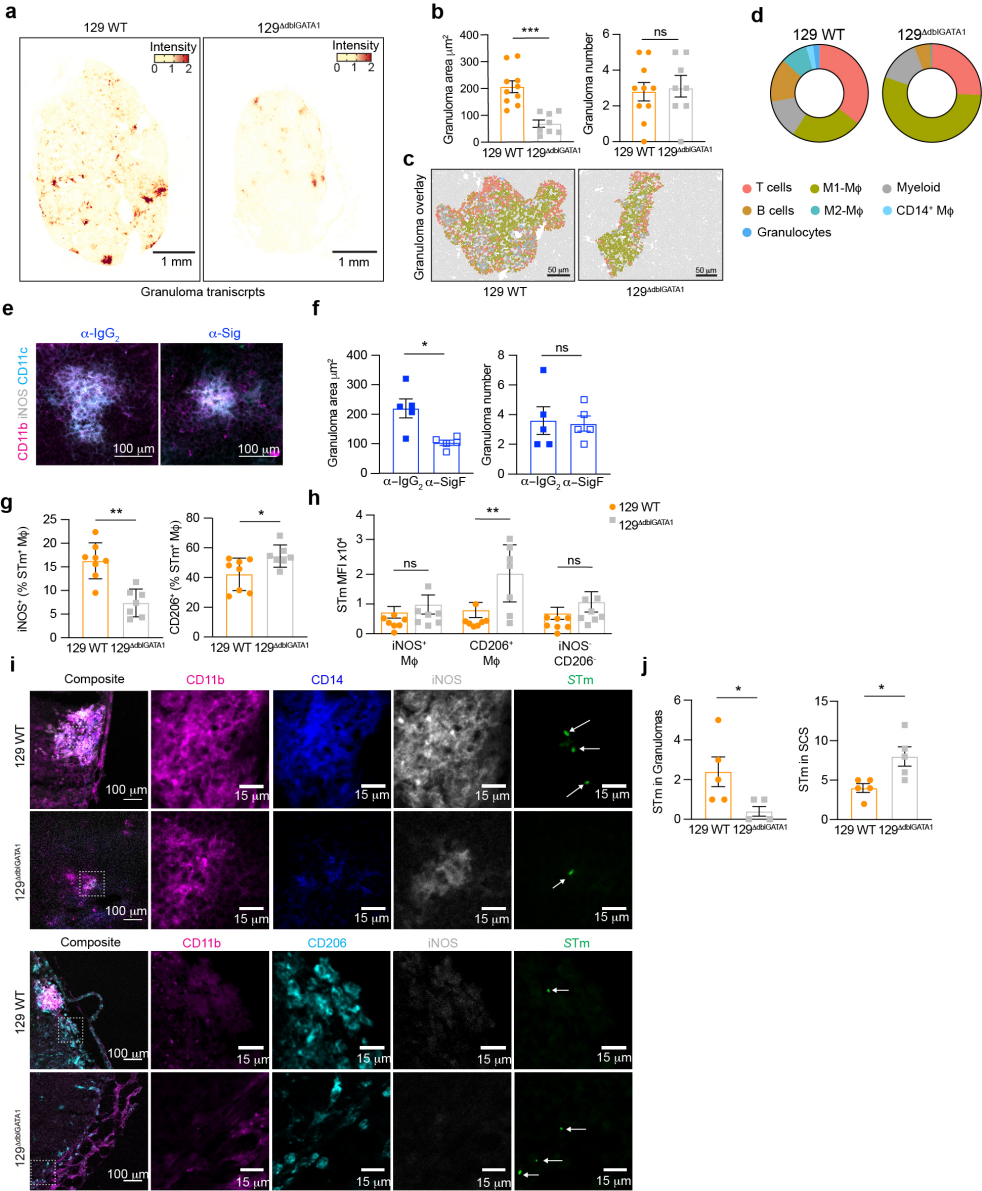
Eosinophils influence MLN granuloma immune cell composition, size, and location of STm-containing macrophages. Eosinophil-deficient 129^DdblGATA1^ mice and 129 WT mice were orally infected with *S*Tm (10^8^ CFUs) for 4 weeks to investigate the impact of eosinophils on macrophage phenotypes and granuloma responses. (**a**) Xenium *in situ* analysis showing representative granuloma-associated transcripts (*Itgam, Nos2, Il1b, Il1rn,* and *Cxcl16*) overlayed on MLN tissue sections from *S*Tm-infected 129 WT and 129^ΔdblGATA1^ mice. Scale bars = 1 mm (n = 6–8 mice in each group from three independent experiments). (**b**) Quantification of average granuloma area and granuloma numbers in *S*Tm-infected 129 WT and 129^DdblGATA1^ mice as defined by immunofluorescence staining of CD11b^+^ iNOS^+^ double positive foci (*n* = 8–10 mice from 3 experiments). (**c**) UMAP clusters overlaid on MLN tissue sections from *S*Tm-infected 129 WT and 129^DdblGATA1^ mice at 4 w.p.i. Representative images from 1 mouse/group. Scale bars = 50 mm. Colors correspond to the UMAP clusters in Extended Data Fig. 6b. (**d**) Donut plots showing the total percentage of the different clusters in granulomas from 129 WT samples and 129^DdblGATA1^. Average data from 6–8 mice per group from 3 independent experiments. (**e**) Representative Immunofluorescence images of MLN tissue sections from *S*Tm-infected anti-IgG_2_ treated mice and anti-Siglec F treated mice showing CD11b (magenta), iNOS (Gray) and CD11c (cyan). Scalebar = 100 mm. (Transient depletion protocol from Fig. 4a). (**f**) Quantification of the average granuloma area and granulomas numbers in MLN from *S*Tm-infected isotype control treated mice and anti-Siglec F treated mice. (**g**) Quantification of *S*Tm stained within iNOS+ and CD206+ macrophages isolated from the MLN of *S*Tm-infected 129 WT and 129^DdblGATA1^ mice (*n* = 5–7 mice in each group from 2 independent experiments). Representative FACS plots in Extended Data Fig. 6i. (**h**) Quantification of *S*Tm Mean fluorescent intensity (MFI) in iNOS+ macrophages, CD206+ macrophages and non-polarized macrophages isolated from the MLN of *S*Tm-infected 129 WT and 129^DdblGATA1^ mice (*n* = 5–7 mice in each group from 2 independent experiments). (**i**) Immunofluorescent staining of MLN tissue sections from *S*Tm-infected 129 WT and 129^DdblGATA1^ mice showing CD11b (magenta), CD14 (blue), iNOS (grey) and STm (green) or CD11b (magenta), CD206 (cyan), iNOS (grey) and *S*Tm (green). Arrows are highlighting *S*Tm localization. Scale bars = 100 mm; insets = 15 mm (*n* = 5 mice in each group from 1 experiment). (**j**) Quantification of *S*Tm observed in granulomas and in the subcapsular sinus (SCS) in *S*Tm-infected 129 WT and 129^DdblGATA1^ mice (*n* = 5 mice in each group from 1 experiment). Data are presented as Means ± SEM and was analysed by Mann-Whitney U-test (B, H, K) * = p < 0.05, ** = p < 0.01, *** = p < 0.001

Macrophage phenotypes and their polarization are shaped by cytokines and chemokines in their microenvironments^33–35^. Noting the distinct differences in *S*Tm-infected macrophages in 129 WT compared to 129^DdblGATA1^ mice, we delved into how these macrophages are distributed in the MLN in these mouse backgrounds. Employing Xenium single-cell *in situ* analysis, we mapped various macrophage transcripts to MLN tissue sections. Our results show that there are diminished M1-granuloma transcript levels in the 129^DdblGATA1^ mice compare to 129 WT mice (Fig. 6a). In addition, there is a striking reduction in granuloma size in the eosinophil-deficient mice compared to wild-type mice as defined by the area of CD11b + iNOS + double positive foci through immunofluorescene staining (Fig. 6b). We further analyzed the cellular compositions within granulomas and in the 50 μm surrounding the granuloma cores (defined by M1-macrophage foci using Xenium single-cell *in situ* analysis) in MLN from 129 WT and 129 ^DdblGATA1^ mice infected with STm for 4 weeks. Our unsupervised clustering identified seven clusters within the granuloma, dominated by M1-like granuloma macrophages. Additionally, granuloma regions contained B and T cells, myeloid cells, M2-like macrophages, *Cd14 +* macrophages, and granulocytes (**Extended Data Fig. 6a, b**). Notably absent in the 129 ^DdblGATA1^ mice were two clusters containing M2-like macrophages and neutrophils, along with a lack of *Cxcl2 + Arg1 +* cells (M2 Mfs) and *Prg2 +* cells (**Extended Data Fig. 6b, c**). These findings were further visualized by spatially mapping these clusters onto MLN tissue sections, highlighting significant shifts in granuloma composition (Fig. 6c, d).

Additionally, our detailed analysis of granuloma-associated transcripts revealed significant differences between the two mouse genotypes. Notably eosinophil-deficient mice had lower expression of key granuloma-associated transcripts such as *Il1b, Il1rn, Cd14* and *Itgam* within granulomas (**Extended Data Fig. 6d, e**). These genetic differences were further substantiated visually through histological and immunofluoresnce staining (**Extended Data Fig. S6f, g**,), illustrating the critical role of eosinophils in shaping the granuloma response during persistent *S*Tm infection.

These findings suggest that eosinophils not only contribute to the granuloma response but also affect granuloma formation and/or maintenance in the MLN. To explore specific effects of eosinophils on granuloma dynamics, mice were infected for 28 days and treated either with isotype control antibodies or anti-Siglec-F antibodies for 14 days as described previsouly to transiently deplete eosinophils (Fig. 4a). At 42 d.p.i, immunofluorescence analysis of tissue sections for granuloma markers revealed that both groups developed granulomas. However, granulomas in eosinophil-depleted mice were significantly smaller, although their numbers remained comparable to those in the control group (Fig. 6e, f). Collectively, our findings underscore the importance of eosinophils in maintaining the abundance of iNOS + macrophages within granulomas, which are critical for containing *Salmonella* infection effectively within the MLN.

Considering the observed decrease in M1-like macrophages in eosinophil-deficient mice (Fig. 5c,e), we next asked whether the intracellular *S*Tm niche is different in 129 WT compared to 129^DdblGATA1^ mice. Flow cytometry analyses revealed a lower percentage of iNOS + macrophages harboring *S*Tm in eosinophil-deficient mice compared to 129 WT mice (Fig. 6g, **Extended Data Fig. 6I**). Moreover, *S*Tm was predominantly found within more permissive, M2-like macrophages and this was notably more pronounced in eosinophil-deficient mice (Fig. 6g). Strikingly, eosinophil-deficient mice dislplayed a higher *S*Tm MFI within CD206 + macrophages, indicating a greater bacterial load in these cells (Fig. 6h). Immunofluorescence microscopy confirmed the flow cytometry findings. For example, there was elevated *S*Tm presence in iNOS + granulomas in 129 WT mice compared to 129^DdblGATA1^ mice (Fig. 6i, j). In addition, we found that *S*Tm primarily associated with CD206 + macrophages in the capsule/SCS area of the MLN in both mouse backgrounds (Fig. 6i, j). However, a significantly larger number of CD206 + cells were associated with *S*Tm in eosinophil-deficient 129^DdblGATA1^ mice (Fig. 6i, j). Taken together or results indicate that eosinophils are crucial for maintaining granuloma structures and that they play a perhaps paradoxical role in creating an environment that supports M1 macrophages which are crucial for containing *S*Tm within MLN during persistent infection.

## Discussion

Our study underscores the intricate challenges associated with persistent intracellular pathogens such as *S. enterica* and *Brucella spp.* which thrive within host cells, complicating treatment strategies, thus prolonging their survival and facilitating spread through asymptomatic carriers^36,37^. This asymptomatic nature magnifies their transmission across populations, representing a formidable public health challenge^38^. Despite robust innate and adaptive immune responses in many infected individuals, these pathogens can remain in infected tissues at low levels for extended periods of time without signs of disease. A pivotal mechanism for this bacterial persistence is the formation of granulomas and the pathogen’s subsequent manipulation of these structures. Our research highlights a novel and unexpected role of eosinophils in regulating granuloma expansion and promoting antimicrobial defenses during persistent *Salmonella* colonization in the MLN. Through the CCL11-driven recruitment, of eosinophils, these cells modulate the myeloid response in the MLN and influence macrophage polarization, thus maintaining a balanced immune environment essential for containing the bacteria and preserving granuloma integrity. Using the Xenium *in situ* platform and analysis tools, we observe that eosinophils contribute to granuloma organization by influencing immune cell recruitment and maintaining localized pathogen control, thereby illuminating the pivotal role of eosinophils in persistent bacterial infections and extending their recognized importance beyond typical type 2 immune responses.

Hyper-eosinophilic responses are highly associated with exacerbated inflammation and subsequent fibrosis in different tissues and often involve elevated levels of CCL11. Studies utilizing *Ccl11*^−/−^ mice or treatments that inhibit CCL11 have demonstrated reduced inflammation in models of asthma and IBD^39,40^. While CCL11’s role in bacterial infections hasn’t been established, its involvement in inadequate control of nematode infections (e.g., *Litomosoides sigmodontis*) suggests a broader immunological impact^41^. In this study, CCL11 neutralization decreased eosinophil numbers and increased STm levels in the MLN without systemic effects, highlighting a targeted role in CCL11-driven bacterial control. This outcome suggests that CCL11’s influence is linked to its ability to direct eosinophil migration to specific tissues, differing from the role of CCL24 (Eotaxin-2) in other systemic organs such as the liver and spleen^42–44^. This tissue-specific eosinophil response in the MLN is noteworthy and needs to be further explored. However, it might be speculated that in different systemic tissues, other cells with immunomodulatory and antimicrobial functions take on the role that eosinophils display in the MLN. Further exploration into the recruitment sources and dynamics of MLN eosinophils during persistent *Salmonella* colonization could offer insights into tissue-specific bacterial defense mechanisms.

Our findings emphasize the contextual and variable antimicrobial roles of eosinophils across different organs and infections. For instance, in *Mtb* infections, eosinophils foster an antimicrobial environment and integrate into granuloma structures^21^. Conversely, in gastrointestinal *H. pylori* infections, eosinophils seem to facilitate colonization^22^. Intrestingly, *Bordetella spp.* infection of the lungs require low eosinophil numbers to colonize and grow, a phenotype that is lost when mice are infected with D*btrS* mutant during semi-acute infection stages^45^. The variabile roles of eosinophils during bacterial infections underscores their ability to adapt their responses based on specific pathogens and environment contexts. *In vitro* studies support that direct antimicrobial actions of eosinophils through release of granules and eosinophil extracellular traps (EETs) are selectively effective, showing potent activity against bacteria like *Pseudomonas aeruginosa* and *Escherichia coli*, but less so against *H. pylori* and *Staphylococcs aureus*^22,46–49^. These antimicrobial actions could also modulate broader immune responses, similar to how neutrophil extracellular traps trigger potent interferon responses^50^. Our data from using eosinophil-deficient 129^ΔdblGATA1^ mice, suggest a diminished antimicrobial action against *Salmonella* in persistent MLN colonization, while acute infection impacts were minimal (**Extended Data Fig. 4a, b**), aligning with other studies in BALB/c mice noting’ limited roles of eosinophils in acute *Salmonella* phases^51^. This distinction between persistent and acute infection roles highlights the complex involvement of eosinophils in immune regulation and pathogen responses.

Beyond their role in antimicrobial defense, eosinophils significantly shape local immune dynamics^52^. Eosinophils modulate T_H_1 responses during *C. rodentium* and *H. pylori* infections and enhance CD8 + T-cell survival in systemic *Listeria monocytogenes* infections^22,24^. Our findings reveal that in the MLN, eosinophils predominantly influence myeloid cell responses, especially impacting neutrophils and macrophages. While eosinophil-deficient MLN show reduced neutrophil counts, *Salmonella* mainly infects macrophages, suggesting neutrophils play a minimal antiimcrobial role in persistent MLN infections. Indeed, previous *in vitro* and *in vivo* studies have indicated that *S*Tm finds a conducive environment in M2-like macrophages, identified by markers such as *Il4ra, Mrc1 and Timp1*^16,53^. In this study, we show that the absence of eosinophils results in fewer macrophages and a decreased presence of iNOS^+^ M1-like macrophages, leading to less structured and smaller granulomas compared to those in 129 WT mice. Interestingly, while both eosinophil-deficient and WT mice harbor *S*Tm primarily within macrophages with an M2-like phenotype that are located in the subcapsular sinus of the MLN, 129 WT mice show a notable proportion of *S*Tm in granuloma-associated cells. This observation suggests that the bacterial containment in WT mice may be enhanced by their localization within a less permissive granuloma niche under semi-chronic conditions. Our data suggest that both 129 WT and eosinophil-deficient mice primarily have a replicative niche in M2-like macrophages within the SCS in the MLN, an area of the MLN essential for trafficking antigens and pathogens during steady-state and gastrointestinal infections^54^. Intrestingly, our data indicate that eosinophil-deficient 129^DdblGATA1^ mice have elevated levels of *S*Tm in the SCS which might suggest that in the absence of eosinophils, *S*Tm get trapped in these areas. This defective response could influence further transport of *S*Tm to the MLN paranchyma and might help explain the global reduction of immune responses observed in these mice (Fig. 5A–E, **Extended Data Fig. 5A-C**) as well as the reduced ability to form and maintain granuloma structures (Fig. 6a–f, **Extended Data Fig. 6e, f**).

Eosinophils, typically observed at the periphery and core of some parasitic granulomas, may excert antimicrobal effects^21,55^. Our findings suggest that in MLN, eosinophils actively modulate the inflammation within granulomas during persistent *Salmonella* infection. This modulation might involve a balance of pro-inflammatory and anti-inflammatory signals mediated by cytokines like IL-4 and IL-13 or by eosinophil granule proteins. Furthermore, PD-L1 expression on eosinophils could be influencing local T cell responses, potentially leading to T cell exhaustion and affecting the granulomas’s immune environment^56,57^. This study is one of the first to illustrate that alterations in eosinophil activity can significantly impact granuloma structure, suggesting eosinophils play a more dynamic role in persistent infection than previously appreciated.

Using Xenium single cell *in situ* analysis, our observations revealed that while granulomas in the MLN predominantly contain macrophages expressing pro-inflamamtory M1 markers, eosinophils are strategically positioned around these formations. This positioning might indicate that eosinophils play a role in providing necessary signals to both granuloma macrophages and other cells surrounding the granuloma, which appear to be crucial for both the maintenance and transition of granuloma structures in the MLN.

Our study highlights the role of eosinophils in mediating antimicrobial effects and supporting MLN granulomas. However, it’s important to note the limitations of our approach. Our Xenium dataset provides spatial resolution at the single cell level, however, our probe selection focuses predominantly on different immune cell populations. To fully understand the interactions between eosinophils and other critical lymph node cells, including various stromal cells, further studies are needed. This bias is reflected both regarding CCL11 production and potential differences in granuloma composition where other types of cells have been implicated to play important roles in different tissues^58,59^.

Further investigations into the dynamics of MLN granulomas at subsequent stages could shed light on whether eosinophils contribute to evolving granuloma characteristics or serve distinct roles during initial stages post-infection. Collectively, our findings underscore the pivotal role of eosinophils in fine-tuning granuloma responses, essential for sustaining effective and normal granulomatous architecture. In summary, this research underscores the importance of a tissue-specific eosinophil response within the MLN, closely integrated myeloid cell dynamics and granuloma formation, during persistent *S*Tm colonization.

## Methods

### Ethics Statement

Experiments involving animals were performed in accordance with NIH guidelines, the Animal Welfare Act, and U.S. federal law. All animal experiments were approved by the Stanford University Administrative Panel on Laboratory Animal Care (APLAC) and overseen by the Institutional Animal Care and Use Committee (IACUC) under Protocol ID 12826. Animals were housed in a centralized research animal facility accredited by the Association for Assessment and Accreditation of Laboratory Animal Care (AAALAC).

### Mice

129×1/SvJ (129 WT), C57BL/6J and B6.129S1(C)-*Gata1*^*tm6Sho*^/LvtzJ (C57BL/6^DdblGATA1^)^60^ were obtained from Jackson Laboratories. 129^DdblGATA1^ mice were generated by backcrossing C57BL/6J^DdblGATA1^ mice with 129×1/SvJ mice for 8 generations. All offspring were genotyped and verified by PCR. Male and female mice (7–9 weeks old) were housed under specific pathogen-free conditions in filter-top cages that were changed bi-monthly by veterinary or research personnel at the Stanford Animal Biohazard Research Facility. Sterile water and food were provided ad libitum.

### Bacterial Strains and Growth Conditions

*Salmonella enterica* serovar Typhimurium (STm) SL1344 and STm SL1344-tdTomato strains were maintained aerobically on LB agar supplemented with 200 μg/mL streptomycin and grown aerobically overnight at 37°C with aeration before infection procedures.

### Method Details

#### Mouse Infection and Treatments

Male or female mice were orally infected with *Salmonella enterica* serovar Typhimurium (STm) at a dose of 1 × 10^8^ CFU in drinking water, as previously described^4,19,61^. Fecal shedding was monitored weekly by collecting a single fecal pellet into 500 μL PBS, following the protocol of Ruddle et al. (2022)^61^. For terminal experiments, mice were euthanized at specified time points using carbon dioxide. To assess tissue bacterial burdens, organs were aseptically harvested and weighed. Each organ was mechanically dissociated, followed by serial dilutions of the homogenate plated on LB agar supplemented with 200 μg/mL streptomycin. Colony-forming units (CFU) were counted after overnight incubation at 37°C to quantify bacterial load.

##### CCL11 Neutralization:

129×1/SvJ mice were orally infected with STm SL1344 as above. After 3 days, infected mice received either anti-CCL11 or IgG2 isotype control antibodies (20 μg/dose) every third day until sacrifice at 28 days (9 doses) (Fig. 2a).

##### Siglec-F Depletion:

Mice were infected with STm SL1344 for 28 days, then received 7 doses of anti-Siglec-F or IgG2 isotype control antibodies (20 μg/dose) every other day, and were sacrificed on day 41 (Fig. 4a).

##### rIL-5 treatment:

Mice infected with STm SL1344 for 28 days received 3 doses of recombinant mIL-5 (20 μg/dose) or PBS every other day, followed by sacrifice on day 35 (Fig. 4f).

The efficacy of all treatments was validated by flow cytometry at sacrifice and data are provided in **Extended Data Figs. 2c** and **4a, b, h, i**)

### Flow Cytometry and FACS sorting

Organs were aseptically harvested into complete media (RPMI + 10% FCS) and kept on ice until further processing. Single-cell suspensions were prepared using a digestion buffer with 1% Liberase and 0.5% DNase I, diluted in complete media, followed by mechanical dissociation using a MACS Dissociator. Cells were incubated at 37°C for 30 minutes, filtered through 70 μm strainers, and centrifuged. Red blood cells were lysed with ACK-lysing buffer (5 min, RT) and washed with FACS buffer (5% BSA, 0.5 mM EDTA in PBS). Cells were blocked with FC-block (1:100 dilution, 15 min), stained with surface markers (**Supplementary Table 1**) (30 min, 4°C), fixed (20 min, RT), and analyzed using a Cytek Aurora cytometer. FACS sorting was performed using a BD FACSAria sorter. Obtained data analysis was performed using FlowJo 10.8.2.

### Microscopy

MLN from uninfected and infected mice were snap-frozen in liquid nitrogen, embedded in OCT, and sectioned at 15 μm thickness. Sections were mounted on double-positive frost slides, fixed in ice-cold acetone (10 min), dried, rehydrated in PBS, and blocked with 10% FBS and 1% BSA in PBS (1 h, RT). Sections were incubated with primary antibodies overnight at 4°C (**Supplementary Table 1**), washed, and stained with conjugated secondary antibodies (1 h, RT) (**Supplementary Table 1**). The slides were washed and mounted, and images were obtained using a Zeiss LSM-700 microscope. Data analysis was performed with ImageJ (Version 2.0.0-rc-54/1.5 1h). For H&E staining, organs were aseptically harvested, washed and fixed in PFA prior to paraffin embedding. 5 μm tissue sections were mounted on slided and stained with hematoxylin and eosin stains per standard procedures and imaged using light microscopy.

### Granuloma quantification

The presence and size of granulomas were identified by immunofluoresence microscopy and defined by CD11b+ iNOS+ double positive foci in the MLN of *S*Tm infected mice. The average granuloma number was quantified by analyzing two sections from the same MLN. The average granuloma area was quantified by measuring the average area of the granuloma core, defined as CD11b+ iNOS+ double positive foci, across the MLN per mouse using ImageJ.

### Cytokine Analysis

Whole tissue lysates were prepared by aseptically harvesting organs, weighing, and lysing tissues in 1 mL lysis buffer with phosphatase and protease inhibitors. Lysates were generated using a MACS Dissociator, and protein concentration was determined via Bradford assay. A pooled sample of 100 μg from five mice per group was loaded onto pre-spotted membranes and analyzed per the manufacturer’s instructions. Membranes were imaged with a BioRad imaging system, and pixel intensity was quantified in ImageJ, normalizing cytokine expression to uninfected controls.

CCL11 and IL-33 tissue levels were measured by ELISA using commercial kits (R&D Systems) on 50 μg of tissue lysate, normalized to tissue weight.

### Xenium in Situ Analysis

#### Probe Selection

100 probes were selected based on single-cell RNA-seq studies targeting myeloid and lymphocyte markers relevant to chronic STm and Mtb infections, as well as eosinophil biology in mice^10,25–27^. Probes targeted specific cell markers and differentially expressed genes obtained from studies of chronic STm, Mtb infections or eosinophil biology in mice. See **Supplementary Table 2** for detailed information of probes used.

#### Tissue Processing

MLN from 16 mice (3 independent experiments) were snap-frozen in liquid nitrogen, OCT-embedded, and sectioned at 10 μm. Sections were fixed in 4% paraformaldehyde (10 min), washed, and dehydrated in an ethanol gradient. Pretreatment was done per Xenium protocol for optimal probe penetration and autofluorescence reduction.

#### Hybridization and Imaging

Probes were hybridized as per manufacturer instructions, and imaging was done on a high-resolution fluorescence microscope. Single-cell spatial resolution was achieved by merging Xenium probe data with the *Tabula Muris* dataset and additional scRNA-seq datasets for *S*Tm and *Mtb* infections^10,25–27^.

#### Data Processing

Xenium data were converted to Seurat objects in R, followed by normalization, batch correction, scaling, and merging. SCTransform normalization was applied, then PCA and UMAP were performed for clustering. Clusters were identified using the Louvain algorithm, optimized for dataset resolution. Heatmaps of marker genes were generated for cluster characterization, and dot plots visualized gene expression profiles.

#### Data Visualization and Granuloma Transcripts

Clusters were overlaid on MLN tissue using Xenium Explorer for spatial localization. Genes of interest were pseudocolored and overlaid on automated segmented cells in Xenium Explorer. For differential expression analysis between 129 WT and 129^ΔdblGATA1^ mice, granulomas and surrounding cells 50 mm outside of the granuloma core (defined by M1-macrophage foci across the MLN) were extracted from Xenium Explorer. Isolated granuloma cell data were converted to Seurat objects in R, followed by normalization, batch correction, scaling, and merging. SCTransform normalization was applied, then PCA and UMAP were performed for clustering. Absolute transcript numbers were isolated from the same cells prior to using a pseudo-bulk approach in R. Samples were Log2-normalized and differentially expressed genes were identified between the two groups using a Log2-fold change >1.41 and p < 0.05 cutt-off.

### Statistics

Data analysis was performed in GraphPad Prism (version 10.2.2) or R using Seurat and Pheatmap. Normal distribution was determined using D’Agostino’s normality test. Statistical analyses included Mann-Whitney U tests, Kruskal-Wallis with post hoc analysis, Mantel-Cox, or χ^2^ tests as indicated in each Figure legend. Significance was defined as p < 0.05.

### Lead Contact and Materials Availability

Further information and requests for resources and reagents should be directed to andwill be fulfilled by the Lead Contact, Denise M. Monack (dmonack@stanford.edu).
